# Walking Along Curved Trajectories. Changes With Age and Parkinson's Disease. Hints to Rehabilitation

**DOI:** 10.3389/fneur.2019.00532

**Published:** 2019-05-24

**Authors:** Marco Godi, Marica Giardini, Marco Schieppati

**Affiliations:** ^1^Division of Physical Medicine and Rehabilitation, ICS Maugeri SPA SB, Pavia, Italy; ^2^Department of Exercise and Sport Science, International University of Health, Exercise and Sports, LUNEX University, Differdange, Luxembourg

**Keywords:** curved walking, aging, Parkinson's disease, freezing of gait, curved walking rehabilitation

## Abstract

In this review, we briefly recall the fundamental processes allowing us to change locomotion trajectory and keep walking along a curved path and provide a review of contemporary literature on turning in older adults and people with Parkinson's Disease (PD). The first part briefly summarizes the way the body exploits the physical laws to produce a curved walking trajectory. Then, the changes in muscle and brain activation underpinning this task, and the promoting role of proprioception, are briefly considered. Another section is devoted to the gait changes occurring in curved walking and steering with aging. Further, freezing during turning and rehabilitation of curved walking in patients with PD is mentioned in the last part. Obviously, as the research on body steering while walking or turning has boomed in the last 10 years, the relevant critical issues have been tackled and ways to improve this locomotor task proposed. Rationale and evidences for successful training procedures are available, to potentially reduce the risk of falling in both older adults and patients with PD. A better understanding of the pathophysiology of steering, of the subtle but vital interaction between posture, balance, and progression along non-linear trajectories, and of the residual motor learning capacities in these cohorts may provide solid bases for new rehabilitative approaches.

## Introduction

Every day we frequently follow circular courses and turns when we move (1). Our inherently unstable bipedal gait (2) requires adaptive control during curved walking (3). Stride length and duration of the stance phase differ between the inner and outer leg (4–6), and the center of mass moves toward the interior of the trajectory (7). This is made possible by appropriate placement of the feet to create a gravity-dependent torque that counteracts the centrifugal force. Central commands would take over the control of locomotion at the expense of the spinal automatisms, very much as it happens for crayfish (8) and giant stick insects, where rotation of the body is accomplished by modulation of ground reaction forces among legs (9). Obviously, the brain generates *ad-hoc* activity that follows the basic laws of physics when moving along circular trajectories, regardless of the number of legs (10).

A thoughtful study of walking and turning in humans had been done more than 140 years ago by Eadweard Muybridge [see (11)]. Foot yaw orientation, body tilt in the frontal plane and pelvis rotation over the stance leg could already be seen in his remarkable photographic freeze frames. Then, non-linear walking trajectories had been almost ignored by scholars and scientist, until Takei et al. published a note (12) about the walking trajectory of young and older subjects on a circular path in darkness.

The interaction between the spinal circuits with the descending command and the adaptation of the body medio-lateral inclination and step length asymmetry to the progression velocity would represent a challenge for the control system. This task requires anticipatory adjustments (13–19) in advance of taking the curve (5, 20–22). It is not implausible that basal ganglia are subjected to an extra load during curved walking, since they operate through two parallel distinct pathways having opposing effects, allowing the execution of movement while gating the antigravity activity of the postural muscles (14, 23). This short review aims to summarize the characteristics of curved walking in young and older adults, and to address gait differences between people with Parkinson's Disease (PD) and healthy subjects. Proposals for an integrated rehabilitation approach in PD are put forward.

## Steering the Body Along Curved Paths

Successful locomotion along curved trajectories requires fine coordination of body segments' movements. Any minor change in the adjustment of the asymmetric step length of the two legs produces dramatic effects in the kinematics, very much as it occurs with minimal changes in the localization of ground reaction forces underneath the feet during the stance phases (6). Therefore, accurate brain control is required for correct rotation of the lower limbs and inversion or eversion of the ankle for successful placement of the foot on the ground (4, 24–31).

During linear walking, the feet create mediolateral impulses at heel strike that symmetrically move the body toward the contralateral limb. In turning, the body is moved toward the interior of the trajectory by both the internal and the external foot (32). In order to exploit gravity, the center of foot pressure during heel strike and toe off is being slightly displaced with respect to its position under linear walking. This creates a mediolateral torque that produces and controls trunk roll tilt and progression along the circular trajectory (6, 33–35), and generates the proper centripetal force to avoid going off on the tangent (4, 5, 27). Appropriate braking of the body fall toward the interior of the trajectory is exerted by the feet at foot-off (6, 36), such as to counterbalance the reaction forces produced at heel strike (37).

In addition to studying walking along circular trajectories, investigators have focused also on the strategies used to navigate sharp turns (26, 30, 38–40). Others have exploited figures of eight trajectories that include both clockwise and counter-clockwise segments (3, 41–43). Consistently, turns imply reduction of progression velocity, placement of the foot in the direction of the new trajectory (25) and lateral translation of the body in addition to body reorientation to align with the new travel direction (15). Foot placement and trunk inclination move the center of mass toward the new direction in the transition stride. The top-down temporal sequence in body segments reorientation slightly changes as turn proceeds (26, 44). This pattern is robust to turning velocity, therefore inherent in the command to turn (45). Gaze redirection accompanies steering, so that visual or oculomotor deficits should be considered when assessing turning behavior (24, 46).

## What Do we Know of the Neural Command for Steering?

Motor tasks employ muscle synergies, i.e., one or more sets of muscles synchronously activated and specific to the task (47). Synergy studies concluded that rectilinear and curvilinear walking share the same motor command; however, fine-tuning in muscle synergies is necessary for circular trajectories, where the kinematic strategy conforms to the physical laws that underpin curved walking while keeping balance (14, 27, 48–50). Courtine and Schieppati (28), using the principal component analysis, found that both straight-ahead and curved walking were low dimensional, and two components accounted for more than 70% of the movement variability. Fine modulation of the muscle synergies underlying the straight-ahead locomotion is sufficient for generating the adequate propulsive forces to steer walking and maintain balance (48).

Bejarano et al. (49) found four muscle synergies for both walking conditions. Muscle activation profiles lasted longer and were larger during curvilinear than straight walking, and more so for the muscles of the limb inside than outside the trajectory. However, several deep muscles responsible for intra- and extra-rotation of pelvis on thigh had not been recorded. The asymmetric activation of these muscles and the amplitude and time-course of the modulation of their activity might configure an additional synergy peculiar to turning. The contribution of the gluteus medius to the trunk orientation during turning should be also considered (51).

The origin of the adaptation of the motor command to the curved path is a matter of speculation. Jahn et al. (52) have described brain activation for imagined straight walking and for imagined walking along a curved path (53). They observed asymmetric basal ganglia activation at turn initiation, enhanced activity in cortical areas associated with navigation, and decreased activity in areas supposed to process vestibular input. These findings point to the complexity of the organization of the command for producing the curved walking trajectory, while the deactivation of certain brain regions may explain why the vestibular input seems to be down regulated during continuous turning while stepping in place (54), similarly to what occurs at the transition between stance and gait (55).

## Sensory Feedback During Walking and Turning

Asymmetric proprioceptive input elicited by vibration of axial (neck and trunk) muscles produces steering and turning (29, 56–59), whereas proprioceptive input from the leg contributes to fine adjustment of the spinal pattern generators for walking (60, 61). Input from axial muscles would play the role of a servo-mechanism, whereby minor asymmetries initiated by asymmetric foot placement (1, 62) would affect the spinal generators to produce the necessary fine changes in leg and foot kinematics accompanying heading changes.

Whether or not continuous walking along a circular trajectory is also favored by a shift in our straight-ahead goes beyond the scope of this short review, but we would note that a shift in subjective straight-ahead occurs after a period of stepping in place on a rotating treadmill (63). In turn, it is not unlikely that a shift in the straight-ahead is produced by the feedback from the muscles producing the rotation of the pelvis and trunk over the standing leg when walking along a curved trajectory or when stepping in place and turning (54, 59). Vision is obviously not necessary for implementing a curved trajectory (5), but the continuous visual field motion would nonetheless favor the fine tuning of the gait synergies underpinning the production of the circular trajectory (24, 64).

These findings suggest that asymmetric proprioceptive input, either produced by the asymmetric kinematics initially produced by the central command to turn or by the artificial activation through muscle vibration, would favor and sustain the steering synergy.

## Aging Affects Steering

Locomotor impairments are an inevitable consequence of aging, and worsen with the associated cognitive decline (65, 66). However, we did not expand here on the issue of the effects of cognitive decline on locomotion. This was a deliberate choice, because this would require an *ad-hoc* article, given the growing number of papers on this complex topic [see the reviews by (67–69)]. Furthermore, the effect of cognitive decline on turning has not received the attention it would deserve, yet.

Walking speed is definitely lower than in the young (70–72), and older adults adopt a more cautious attitude when steering (73–75). Normally, during the swing period, young subjects reverse the fall of the center of mass before foot-contact by active braking via activation of the triceps of the stance leg (76, 77). The control of this braking phase is impaired in older adults, and the braking phase is compensated for by reducing the step length (78). Neurological (PD, cerebellar syndromes, neuropathies, hemiparesis, dementia) and non-neurological conditions (cardiovascular and respiratory) contribute to gait problems (79, 80).

**Figure 1:**
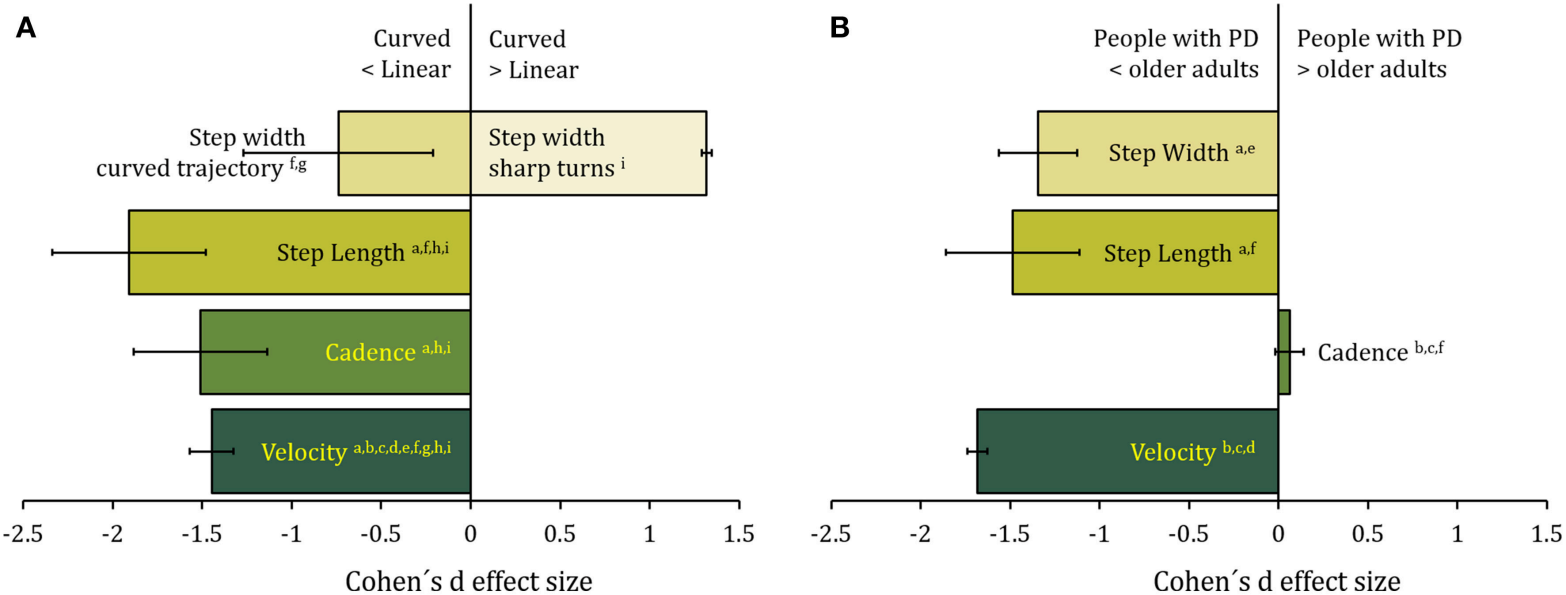
**(A)** The effect of the trajectory (curved with respect to linear) on spatiotemporal gait variables in older adults. Data are the sample-size-weighted mean of Cohen's d effect-size (ES) of the illustrated variables [calculated from ^a^(82); ^b^(41); ^c^(83); ^d^(42); ^e^(87); ^f^(86); ^g^(88); ^h^(85); ^i^(84)]. Negative values in the x-axis represent a decrease in the variables in curved compared to linear walking. Error bars represent 95% confidence intervals. There is an overall decrease in step length, cadence, and step width during protracted curved path. On the contrary, during sharp turns, step width is increased in the older adults. **(B)** The effect of Parkinson's disease on walking along curved trajectories. Data are the sample-size-weighted mean of Cohen's d effect-size of the illustrated spatiotemporal gait variables [calculated from ^a^(89); ^b^(82, 90) ^d^(91); ^e^(92); ^f^(85)]. Negative values in the x axis represent a decrease in the variable values in patients with PD compared to age-matched controls. Error bars represent 95% confidence intervals. Step length and width decrease, while cadence is unaffected. Velocity is diminished mainly because of reduction in step length.

In young adults, curved walking significantly decreases walking speed (by about 15%) and stride-length (more so in the leg inner to curvature), whilst cadence is barely diminished (5, 27). In older adults whose linear gait speed is within the limits of normality (81), curved gait speed diminishes by about 20%, with minor differences across the studied cohorts (42, 82–86) (Figure 1A). In older adults with poor mobility and linear walking speed below the 0.90 m/s, the reduction in gait speed between linear and curved walking is of about 15% (41) and 5% (87). Likely, frailty and balance-related anxiety (93) reduces speed during linear walking to such low values that the time necessary for the added coordination of posture and progression during curved walking becomes proportionally negligible.

Cadence diminishes by <10% (84–86) for older adults during curved when compared to linear walking. Step length is also reduced by <20% (82, 84). While velocity, cadence, and step length changes are common to different types of turning, step width behaves differently between curved walking and sharp turning. Older adults increase their step width during the transition phase from linear to curved path (88). On the contrary, for protracted curved path, step width is narrow (89, 94) and it decreases until 30% (84). Slow anticipatory adjustments may play a role, as shown by abnormal turning pattern in older adults with balance deficits when the command to turn is unexpected (95).

The variability of step length, cadence, and step width increases during curved walking (84, 89, 91, 96). However, in older people who have not fallen, a moderate amount of step variability is required to adapt to situations that challenge postural control (97, 98), while too little variability is associated with fall history in older adults (99). This could explain why increased spatial variability during curved path identified subjects with better motor skills of walking (86).

Additionally, older adults who frequently fall display a reduced turning angle variability compared with non-fallers (100), denoting a lack of dynamic balance skills necessary to seamlessly modulate turning angles while maintaining balance (101). It seems that the ability to vary step length and step width enables both smooth continuity of the center of pressure path and energy-efficient navigation of curves (86).

The multisegmental control has been studied during planned turning on the spot (102–104). Older adults tend to reorient their head, shoulder and pelvis simultaneously, followed by foot displacement, regardless of visual condition. The command to implement curved walking implies modification of all the fundamental spatio-temporal variables of gait (105), more so in older adults who frequently fall compared with non-fallers (100). Interestingly, multiple fallers show a simplified turning pattern to assist balance control (106). Moreover, changes in timing and sequencing of segment reorientation produce earlier anticipation of turns (102, 107). Activity in prefrontal cortex is increased in older compared to young adults (108) suggesting a higher cognitive cost for gait control even during linear walking. In complex walking, known to depend on appropriate executive function and balance control (109), obstacle negotiation further increases prefrontal activity (110).

These studies point to definite changes in the spatio-temporal gait variables between linear and curved walking. The increased variability in the curved gait kinematics can be the result of a coordination disorder but represents kind of a safety factor for reducing the risk of falling. Adaptive strategies for turning might be driven by the effort to diminish the cognitive cost associated with turning.

## Parkinson's Disease Problems During Steering

In patients with neurological disorders, impaired locomotion is a frequent and serious symptom (111). Gait and balance impairments are typical of patients with PD (112, 113). Impaired locomotion is often associated with reduced ability to brake the fall of the body in late stance compared to matched controls (114). This may be a sign of poor coordination between trunk and lower limb, and forces patients to take short steps. A study of muscle synergies has identified a reduced number of synergies during straight walking (115), providing evidence that control of gait is less complex or flexible in patients with PD. Whether this can affect the coordination between trunk and lower limb muscle, and whether synergies are further affected by turning, remains to be established (115).

In neurological patients, additional problems emerge during curved compared to linear trajectories (116–118). Patients with PD are no exception, and all of them are indeed critically challenged by curved walking (119–123), in spite of their diverse phenotype and motor symptomatology. There is a strong indication, based on functional MRI, that patients with PD have reduced activity in the globus pallidus and enhanced activity in the supplementary motor area during imagined walking and turning (124). Functional near infrared spectroscopy shows higher prefrontal cortex activity in the patients compared to healthy subjects (125). Interestingly, prefrontal activation during obstacle negotiation was increased more than during dual task walking (126).

The changes observed in curved compared to linear walking are usually non-negligible. Kinematic analysis demonstrated *en-bloc* rotation of axial segments in patients with PD (127–131). Coordinated axial muscle activation seems to be particularly affected (132). This has been repeatedly shown and assessed quantitatively (133–138). The *en-bloc* rotation of axial segments in patients with PD contrasts with the lower extremity muscle activation pattern that appears to be overall normal (131). Huxham et al. (139) have shown that stride length reduction appears to contribute more than downscaled rotation amplitude to inefficient turning in patients with PD, possibly because of reduced axial mobility. Compared to straight walking, during curved walking speed diminishes by about 20% (90) and by about 35% (82, 91) in less and more affected patients, respectively. Cadence also diminishes by more than 5% and step length diminishes by about 20% (82, 89) (Figure 1B). In contrast to age-matched controls, patients with PD turn with narrower steps (89, 92, 122).

Greater variability of the temporal gait parameters is detectable during curved but not linear walking, over and above the increased variability exhibited by healthy subjects (6). Problems in turning and curved walking are detectable even at an early stage of the disease, and they persist while on-phase (92, 140). Most interestingly, spatio-temporal gait pattern and variability during curved walking are abnormal even in well-treated, well-functioning patients with PD, exhibiting no change in speed in straight-line walking compared to age-matched healthy subjects (85). Anxiety should be considered when evaluating walking performances, since well-treated patients with fear of falling spend more time turning, both in the laboratory and at home (141). This would be connected to the effect of temporal pressure on the control of medio-lateral stability, a critical issue when changing direction (142, 143).

All in all, it seems safe to posit that patients with PD suffer from specific disorders of curved walking and turning, which may not be obvious during straight walking. Again, it may not be immediately clear whether some abnormalities are direct consequences of the neuronal damage or, at least in part, the outcome of a long-duration adaptive process.

## Freezing in People with Parkinson's Disease

Freezing can occur during all types of gait and is related to the increased stride-to-stride variability (144). However, it is most common during turning (22, 145–153) and is a major cause of falling in these patients (154). Turning during daily activities is more compromised in patients with than without freezing (155).

The increase in freezing events during turning may be due in part to the asymmetric nature of the task and the necessary anticipatory adjustment for ensuring postural stability along the medio-lateral direction. The temporal and spatial asymmetry of steps during turning represents a more complex control problem than forward walking (30, 121, 149, 156, 157), as suggested by increased activation in prefrontal areas accompanying freezing before anticipated turns (125). Perhaps, these changes accompany and compensate for the structural and functional alterations in the brain stem centers for locomotion (158).

Neck and axial rigidity during turning may reduce forward progression (159, 160). Patients take shorter turns with smaller turn angles and more steps and exhibit larger variability with respect to controls (161). Freezers, irrespective of freezing episodes, adopt a narrower step width compared to controls and non-freezers during turning (89, 91).

Problems in fast axial turning appear when stepping is performed on a narrow base (160, 162, 163). Freezing episodes are more frequent at sharp turns (91) and turning in place (164), indicating that problems exist both in adjusting the anticipation to the intended trajectory and in controlling body segment coordination and balance during rotation on the spot, regardless of the speed of turning and the severity of the disease (22, 94). Again, this speaks in favor of a delayed preparation for the change in walking direction.

Anticipatory adjustments are indeed abnormal in patients with PD (165–167) including eye and head anticipatory movements for exploration (53) and movements to correct a lateral disequilibrium (168, 169). Plate et al. (170) have confirmed that anticipatory adjustments are slower and of smaller amplitude, in keeping with the overall bradykinesia of the patients. Interestingly, anticipatory postural adjustments are not followed by coordinated steps (171). Anticipatory adjustments associated with gait initiation may not be strictly abnormal *per se* in patients with respect to age-matched subjects (169, 172), but may be impaired in people with freezing of gait compared to those without freezing (173). All in all, these findings are in keeping with the hypothesis that freezing would depend on the sheer control of the trunk rotation over the standing leg (54, 85).

Freezing is elusive. However, any voluntary human movement requires a complex array of in-series and in-parallel processes, from anticipatory postural adjustment to ongoing feedback-related correction. No wonder some peculiar interaction of aberrant events in patients with PD, from cognitive to reflex nature, can finally produce what we call freezing, in our case of gait, but not necessarily limited to gait [see e.g., (174)].

## Rehabilitation of Curved Walking

The ability to turn, above all in restricted spaces, is very important in autonomy maintenance in everyday life. Furthermore, falls during turning result in more hip fractures than falls during linear gait (175). Therefore, rehabilitation of curved walking and turning may be meaningful since turning impairments are not improved by dopaminergic medication (92). Exercise has potential to improve many clinical issues in patients with PD such as strength, balance, walking, and quality of life (176–179). A recent review, not centered on curved walking (180), confirmed the benefits of physiotherapy in most outcomes over the short term. However, most of the observed differences between treatments were considered of minimal clinical importance.

Rehabilitation should address the critical steps in producing successful steering, the anticipatory adjustments in preparation to turn and direct steps along the curved trajectory, the rhythmic and regular production of steps, axial mobility and head-trunk coordination, and the coordinated activity of the pelvic muscles producing intra- and extra-rotation of the lower limbs. Anticipatory postural adjustments can be possibly enhanced by focused appropriate rehabilitation in older adults (181). This is significant in consideration of the relevance of the delayed release of anticipatory adjustments in these patients (92, 167).

The use of linear treadmill for the rehabilitation of gait in patients with PD has produced modest but significant effects on gait speed and stride length (182–185), indicating that these patients can be trained on treadmill and show some improvement. An earlier study with the use of the rotating treadmill in two patients showed that a short period of walking on the rotating treadmill reduces freezing episodes (186, 187). However, training curved walking by a rotating treadmill produced no improvements in gait or turning after 5 days of training (90). A later study, administering a progressive training with augmenting rotation velocity and trial duration through 10 daily sessions, showed instead a significant benefit on walking velocity along a circular path (188). In the latter study, before and at the end of the treatment, all patients walked over ground along linear and circular trajectories, and the velocity of walking bouts increased post-treatment, more so for the circular than the linear trajectory. Therefore, in spite of their known problems in attentional resources and cognitive strategies (189), patients can learn to produce turning while stepping on the rotating treadmill and this capacity translates into improved over ground curved walking. These findings strengthen the conclusions of independent reports by Cheng et al. (190, 191), employing a complex walking program (S-shaped, figure-of-8, square- and oval-shaped paths) or a rotating platform.

Notably, the synergies responsible for maintaining a fixed body orientation in space while stepping on the rotating treadmill (45) are the same that are put in place when stepping in place while voluntarily turning (54). Prolonged stepping in place and turning produces in normal subjects an after-effect consisting in a long lasting spontaneous turning on the spot (eyes closed), likely created by adaptation to the continuously activated somatosensory channel (54, 59, 192). Time decay of angular velocity, stepping cadence, and head acceleration were remarkably similar after both conditioning procedures [voluntarily stepping-and-turning and the so-called podokinetic stimulation by the rotating treadmill (193)]. Therefore, stepping in place while voluntarily turning could be administered *in lieu* of the rotating platform for training the turning task in patients with PD. This treatment could be as successful as the rotating platform for the purpose of exercising the coordinated movements underpinning turning and of training curved walking. In this connection, it is worthwhile to mention that Aman et al. (194) have shown the effectiveness of proprioceptive training in improving sensorimotor function, and Elangovan et al. (195) proved that proprioceptive training is indeed effective in patients with PD.

The responses of patients with PD to vibration of postural muscles are largely normal, even if they show abnormal transient postural responses to vibration-off, a sign of impaired sensory reweighting in balance control (196). This is reminiscent of their inappropriate response to light-off when balancing on a movable platform (197). Similarly, alternate trains of postural muscle vibration promote cyclic body displacement in standing patients much as in age-matched subjects (198), showing that these patients can integrate and exploit the vibratory proprioceptive input to produce postural oscillations comparable to those occurring during walking. Moreover, vibratory stimulation of trunk muscles significantly increases stride length, cadence and velocity in both patients and healthy subjects (199). As far as curved walking is concerned, it is notable that, in healthy subjects, vibration of trunk muscles interferes with the above mentioned podokinetic aftereffect by enhancing—or reducing—body rotation velocity depending on the vibrated side (59). The summation of vibration and podokinetic effect speaks for the capacity of the proprioceptive input from the trunk and from the pelvis muscles to affect steering by modulating the activity of the responsible brain centers through a common mechanism. These interventions (unilateral axial muscle vibration, stepping on the rotating treadmill, voluntarily stepping-and-turning) might be considered when planning a training protocol aimed at rehabilitating gait with emphasis on curved walking.

Overall, it seems that interventions based on the new evidence about planning, organization, and execution of curved walking and associated postural control represent a promising rehabilitation approach. We are now ready for undertaking large-scale studies that address the effects of sensory feedback and of stepping-and-turning task repetition on steering and turning capacities in older subjects and patients with PD, also considering patients with typical PD and atypical parkinsonism [see (200)].

## Conclusions

The findings summarized here suggest that the turn-related command operates by fine modulation of the phase relationships between the tightly coupled neuronal assemblies that drive motor neuron activity during walking. Aging and Parkinson's disease seem to affect steering by slowing the expression of this modulation and simplifying the neural command by coupling subroutines. Older adults and more so patients with PD are compelled to modify the gait pattern, reduce some spatiotemporal variables when facing curvilinear trajectories, and adopt *en-bloc* rotation of axial segments. This strategy allows the CNS to manage a less complex task, even if it does not fully protect these subjects from the risk of falling, likely because of persisting poor posturo-kinetic coordination. The cost of the new control mode, implying a newly designed integration of vestibular, proprioceptive, and visual information for equilibrium control during curved walking, turns out to be substantial in patients with PD, and would lead to freezing and frequent falls. More research is needed on rehabilitative interventions to train voluntary or treadmill-induced body rotation and on their potential of improving walking performance during curvilinear paths.

## Author Contributions

All authors listed have made a substantial, direct and intellectual contribution to the work, and approved it for publication.

### Conflict of Interest Statement

The authors declare that the research was conducted in the absence of any commercial or financial relationships that could be construed as a potential conflict of interest.
